# Electronic Tongue Coupled to an Electrochemical Flow Reactor for Emerging Organic Contaminants Real Time Monitoring

**DOI:** 10.3390/s19245349

**Published:** 2019-12-04

**Authors:** Cátia Magro, Eduardo P. Mateus, Juan M. Paz-Garcia, Susana Sério, Maria Raposo, Alexandra B. Ribeiro

**Affiliations:** 1 CENSE, Department of Sciences and Environmental Engineering, NOVA School of Science and Technology, NOVA University Lisbon, Caparica Campus, 2829-516 Caparica, Portugal; epm@fct.unl.pt; 2 Department of Chemical Engineering, Faculty of Sciences, University of Malaga, Teatinos Campus, 29010 Malaga, Spain; juanma.paz@uma.es; 3 CEFITEC, Department of Physics, NOVA School of Science and Technology, NOVA University Lisbon, Caparica Campus, 2829-516 Caparica, Portugal; susana.serio@fct.unl.pt (S.S.); mfr@fct.unl.pt (M.R.)

**Keywords:** triclosan, electrochemical treatment, real time monitoring, layer-by-layer technique, sputtering technique, electronic tongue, sensors

## Abstract

Triclosan, which is a bacteriostatic used in household items, has raised health concerns, because it might lead to antimicrobial resistance and endocrine disorders in organisms. The detection, identification, and monitoring of triclosan and its by-products (methyl triclosan, 2,4-Dichlorophenol and 2,4,6-Trichlorophenol) are a growing need in order to update current water treatments and enable the continuous supervision of the contamination plume. This work presents a customized electronic tongue prototype coupled to an electrochemical flow reactor, which aims to access the monitoring of triclosan and its derivative by-products in a real secondary effluent. An electronic tongue device, based on impedance measurements and polyethylenimine/poly(sodium 4-styrenesulfonate) layer-by-layer and TiO_2_, ZnO and TiO_2_/ZnO sputtering thin films, was developed and tested to track analyte degradation and allow for analyte detection and semi-quantification. A degradation pathway trend was observable by means of principal component analysis, being the sample separation, according to sampling time, explained by 77% the total variance in the first two components. A semi-quantitative electronic tongue was attained for triclosan and methyl-triclosan. For 2,4-Dichlorophenol and 2,4,6-Trichlorophenol, the best results were achieved with only a single sensor. Finally, working as multi-analyte quantification devices, the electronic tongues could provide information regarding the degradation kinetic and concentrations ranges in a dynamic removal treatment.

## 1. Introduction

Nowadays, both water quality and availability are under stress. The global population is expected to exceed nine-billion by 2050, where 70% will be, living in urban areas [1]. This rising demand for water, together with poor or inefficient wastewater management and limited disposal strategies with minimal treatment practices, increases the need of sustainable tools to assure the quality, monitoring, and prosperity of a healthy population and environment [2]. Emerging organic contaminants (EOCs) are defined as “chemical substances that have no regulation and are suspected to negatively affect the environment or whose effects are unknown” [3,4]. Among EOCs, antibiotics, disinfectants, and antiseptics are especially relevant, as thousands of tons are yearly consumed worldwide in medicine, agricultural, and as daily consumer products [5]. Furthermore, the reported data show that conventional wastewater treatment plants are inefficient in their elimination [6,7,8,9,10,11,12]. Triclosan (TCS or 2,4,4′-Trichloro-2′-hydroxydiphenyl ether) is a broad antimicrobial agent, with low water-solubility (more susceptible to bio-accumulation) and that has been used for more than 50 years as an antiseptic, disinfectant, or preservative in clinical settings and several consumer products, i.e., toothpastes, cosmetics, and plastics. On the basis of the available studies, it is now accepted that it has extensive environmental and health effects, also being toxic to water living organisms due to its photodegradation to chlorodioxins [13,14]. TCS has been detected in wastewater treatments plants and surface waters [15,16]. Moreover, TCS derivatives have also been found, such as the metabolite methyl-triclosan (M-TCS) [17], which is considered to be more persistent [18] and TCS by-products, such as 2,4-Dichlorophenol (DCP) and 2,4,6-Trichlorophenol (TCP), which pose a health risk to humans and are recognized as persistent priority pollutants in the United States, Europe, and China [19].

Innovative green tools for monitoring the degradation processes are currently in the spotlight. Sensors, as compared to traditional sampling and analysis procedures, can provide fast response on the output data in a continuous, safe, and cost-effective way, and therefore may play a role in monitoring contaminants’ dynamics [20]. Among sensors, the electronic tongue (e-tongue) is gaining special attention for liquid matrices. An e-tongue is a multi-sensory system, which formed by an array of sensors with low-selective thin film layers or sensorial layers, and combined with advanced mathematical procedures for signal processing based on pattern recognition and/or multi-variate data analysis [21]. E-tongues have proved to be suitable devices for monitoring aqueous environmental matrices contaminated with EOCs [22,23]. Some examples are Campos et al. (2012) [24], who developed a voltammetric e-tongue [set of noble (Au, Pt, Rh, Ir, and Ag) and non-noble (Ni, Co, and Cu) electrodes to the prediction of concentration levels of soluble chemical oxygen demand, soluble biological oxygen demand, ammonia, orthophosphate, sulphate, acetic acid, and alkalinity from influent and effluent wastewater. Years later, Cetó at al. (2015) [25] used a voltammetric bio e-tongue for simultaneous monitoring of catechol, m-cresol, and guaiacol mixtures in wastewater. Thus, one of the most interesting aspects that motivate the development of e-tongues is their potential for real-time parallel monitoring of multiple species and multi-analyte determination in a single sample analysis [26,27]. The working electrodes in the e-tongues array can be covered with films (coatings), which improves the sensitivity of the electrical measurements. The ability to tune the composition of nanostructured thin films allow for an improvement in the sensor’s intrinsic (chemical or physical) properties for sensing applications. The layer-by-layer (LbL) nano-assembly technique, where the Brazilian group was the pioneer in the subject [28,29,30,31], is a flexible, easily-scalable, reproducible, and versatile approach that allows for the precise control of the coating thickness, composition, and structure. This nano-assembly technique is a powerful tool for the incorporation of a wide variety of coating types, such as polyelectrolytes. The type of thin film or sensorial layer chosen is a critical step for the accomplishment of a reliable qualitative and quantitative device. In a preliminary study, Magro et al (2019) [32] found that the LbL films prepared with polyethylenimine (PEI) and poly (sodium 4-styrenesulfonate) (PSS) build up with five bilayers were suitable for TCS detection in wastewater. However, the polyelectrolyte layers of these thin films may desorb when they are immersed in solutions below pH = 7. According to Zhu et al. (2003) [33], even if the PEI degree of ionization is strongly pH dependent and the PSS is a strong polyelectrolyte, with a pKa near 1, it can be influenced by solution pH due to the presence of a sulfonate group in its chemical structure and furthermore. Studies on layer-by-layer films that were prepared from aqueous solution with pH below 7 revealed that the PSS is completely ionized [34,35]. In this regard, thin films of TiO_2_ and ZnO build-up with the sputtering technique can also be considered as sensorial layers. These films proved to be efficient for the detection of molecules with phenolic rings and they presented high mechanical stability under pH fluctuations [36,37,38]. This study aimed to achieve a customized e-tongue device to follow real-time degradation and quantification of TCS and its derivative by-products in an electrochemical flow reactor (EFR) treatment. The EFR, which mimics the secondary clarifier in a wastewater treatment plant, was considered to be the most appropriate choice to couple with a monitoring real time tool device, due to its potential to operational implementation and final matrix conditions (non-aggressive for thin film layers, e.g., pH effluent around 7.6). 

To attain this proof-of-concept goal: (1) an array of thin films were prepared by LbL and sputtering techniques and were characterized by a field emission scanning electron microscope, in order to understand the device response for each EOCs; (2) the impedance electrical properties of these films when immersed in effluent spiked with EOCs’ were acquired; (3) “calibration curves”, to individually distinguish each EOCs were the base to train the customized e-tongue; and, (4) PCA results from the e-tongue attached to a dynamic EFR were analysed to apprehend the potential for working as a monitoring real time tool for EOCs’ degradation detection and semi-quantification.

## 2. Materials and Methods

### 2.1. Chemical and Standards

TCS (99%), M-TCS (99%), DCP (98%), and TCP (98%) were purchased from Sigma-Aldrich (Steinheim, Germany, Table S1). Individual stock solutions for calibration purposes were prepared with 1000 mg/L in methanol and stored at −18 °C. The methanol, acetonitrile, acetone, and formic acid used were from Sigma-Aldrich (Steinheim, Germany) in gradient grade type. Water (Type I) was from a Millipore system (Aqua Solutions, Bedford, MA, USA).

The sensor devices were purchased from DropSens (Llanera Asturias, Spain) and they were formed by either a BK7 glass solid substrate with deposited interdigitated electrodes comprising 125 “fingers” each or by a ceramic solid substrate with deposited gold interdigitated electrodes comprising eight “fingers” each. The supports’ dimensions were 22.8 × 7.6 × 0.7 mm and each “finger” had 10 or 200 µm of width, which was the same spacing between them. PEI and PSS from Sigma-Aldrich (St Louis, MO, USA) were the chemicals used to prepare the sensing layers on the interdigitated electrodes. The used gases argon, oxygen, and nitrogen had ≥ 99.9% of purity. The used effluent, pH (7.6 ± 0.5) and conductivity (1.6 ± 1.0), was the liquid fraction that was collected in the secondary settling tank at a wastewater treatment plant (Lisbon, Portugal).

### 2.2. Methods

#### 2.2.1. Emerging Organic Contaminants Extraction and Quantification: Chromatography Approach

The extraction of EOCs in the effluent was performed by solid-phase extraction (SPE) while using Oasis HLB (200 mg, 6 mL) from Waters (Saint-Quentin-En-Yvelines Cedex, France). The SPE cartridges were conditioned by washing with 3 × (6 mL) of methanol, followed by re-equilibrium with 3 × (6 mL) of Milli-Q water. For EOCs enrichment, the samples were acidified to pH = 2 before extraction using nitric acid. The 200 mL aqueous samples passed through the cartridge at a flow rate of approx. 10 mL/min by applying a moderate vacuum, followed by a dried period of approx. 3 min by vacuum. The retained EOCs were eluted sequentially with 2 × (4 mL) of methanol and 1 × (4 mL) of acetone. 

The EOCs determination was performed in an Agilent 1260 Infinity II high-performance liquid chromatography (HPLC) that was equipped with a quaternary pump and auto-sampler and a diode array detector (DAD)/fluorescence detector 1100 Series. An EC-C18 column (InfinityLab Poroshell 120 High Efficiency, 100 mm × 4.6 mm; 2.7 µm with Column ID, Agilent, Santa Clara, CA, USA) was used. All of the HPLC runs were performed at a constant flow of 1.5 mL/min in gradient mode, with the oven set to 36 °C. A mixture of acetonitrile, Milli-Q water and formic acid was used as eluents (A: 5/94.5/0.5% and B: 94.5/5/0.5%) with a gradient of 60% of B (0–2 min), followed by 97% of B (2–3.5 min) and 98% of B until 5 min “Calibration curves” were performed in the range between 0.5 and 20.0 mg/L. The limits of detection and quantification were, respectively, 0.7 and 2.0 mg/L for TCS, 1.3 and 3.9 mg/L for M-TCS, 0.7 and 2.0 mg/L for DCP, and 1.0 and 3.0 mg/L for TCP. The recovery tests were made with fortified effluent for 1 h of contact time (30 min of slow agitation). The recovery percentages were between 62% and 120% in all cases.

#### 2.2.2. Emerging Organic Contaminants Quantification: Customized Electronic Tongue Concept

**Figure 1:**
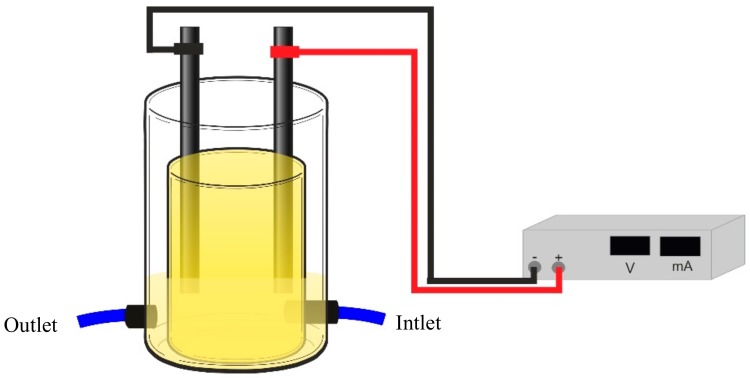
Electrochemical flow reactor used to attach the e-tongue.

An array of sensor devices with different thin films that were prepared by LbL and sputtering techniques deposited onto solid substrates with gold interdigitated electrodes were coupled to an EFR (Figure 1). The e-tongue (Figure S1 for the information of the five sensors that composed the e-tongue) was used to detect the TCS, M-TCS, DCP, and TCP degradation in the effluent.

The LbL thin films were prepared with PEI and PSS polyelectrolytes by the LbL technique [28]. Accordingly, thin films of PEI/PSS that were deposited onto solid support with gold interdigitated electrodes were obtained by adsorbing alternate layers of electrically charged polyelectrolytes at solid/liquid interface, carefully washing with water (Type I) the already adsorbed layers after immersion in the polyelectrolyte solution to remove the polyelectrolyte molecules that were not completely adsorbed. The polyelectrolytes solutions were performed with a polymeric concentration of 10^−2^ M diluted in water Type I, produced with a Millipore system (Bedford, MA, USA). The adsorption time period of each layer (immersion time in each polyelectrolyte solution) was 30 s and the thin film was dried [39] while using a nitrogen flow after the adsorption of each layer. The aforementioned sequence was repeated five times in order to obtain a film with five bilayers, denoted as (PEI/PSS)_5_.

Monolayered films: TiO_2_, ZnO and bilayered film: TiO_2_/ZnO (being ZnO the upper layer) were deposited at room temperature onto gold interdigitated electrodes glass substrates (22.8 × 7.6 × 0.7 mm), by DC (voltage source, Huttinger PFG 10000) reactive magnetron sputtering in a custom-made system. Tianium and zinc discs (Goodfellow, 99.99% purity) with 64.5 mm of diameter and 4 mm of thickness each were used as the sputtering targets. A turbomolecular pump (Pfeiffer TMH 1001) was used to achieve a base pressure of 10^-4^–10^-5^ Pa (before introducing the sputtering gas). Before the sputter-deposition step of the films, a movable shutter was interposed between the target and the substrates. The target was pre-sputtered in the Ar atmosphere for 2 min to clean the target surface. The target-to-substrate distance was kept constant at 100 mm. Gases in the system were pure Ar and O_2_ and needle valves separately controlled their pressures. TiO_2_ and ZnO depositions were both carried out in 100% O_2_ atmosphere. For the TiO_2_ film the total pressure was kept constant at 2 Pa, the sputtering power was 530 W, and the deposition time was 25 min. In the case of ZnO film, the total pressure was fixed at 4.8 Pa, the sputtering power was 300 W, and the deposition time was 30 min. TiO_2_/ZnO bilayered films were prepared while using the aforementioned deposition conditions for TiO_2_ and ZnO. No external substrate heating was used during the deposition. The substrate temperature was measured by a thermocouple passing through a small hole in a copper piece that was in contact with the substrate. During the deposition process, the sample temperature increased up to 60 °C due to the plasma particle bombardment of the substrate. The characterization of the thin films thickness and morphology was performed by a field emission scanning electron microscope (FEG-SEM JEOL 7001F) operating at 15 keV. A gold thin film was coated on the films surface before SEM analysis to charge build-up prevention. The images of the cross section allowed for the estimation of the films’ thickness. Therefore, the measured thickness is given by the trigonometric equation since the positioning of the sample has a slope relative to the axis of incidence of the electron beam which can be deduced through the geometry involved. The indicated correction was calculated while using Equation (1).

(1) $$d_{SEM}=\;\frac{d_{obs}}{\cos\alpha }$$

where the *d_SEM_* is the real thickness, *d_obs_* is an average of the measured thickness values estimated from the cross-section images and α is the beam incident angle (20°).

The electrical analysis of the aqueous matrices was performed by measuring sequentially the impedance spectra of each sensor device when immersed (about 3 min each) in effluent with different EOCs concentrations, while using a Solartron 1260 Impedance Analyzer (Solartron Analytical, AMETEK scientific instruments, Berwyn, PA, USA) in the frequency range of 1 Hz to 1 MHz, while applying an AC voltage of 25 mV. The impedance data was collected from the SMaRT Impedance Measurement Software (AMETEK scientific instruments, Berwyn, PA, USA). Calibration solutions were performed at 25 °C and then prepared while using effluent spiked with EOCs concentrations in the degradation range that were expected in the electrochemical flow reactor (0 to 0.8 mg/L). For all measurements a blank standard (0 mg/L) was used. For the electro-degradation monitoring, every 15 min (total time of treatment 120 min) was assembled an aliquot to be measured for each sensor, in the impedance conditions that are described above.

#### 2.2.3. Data Analysis

Principal component analysis (PCA) was carried out regarding the normalized Z-score normalization, $z\;=\;\frac{x-\;\mu }{\sigma }$ impedance spectroscopy data (capacitance, impedance, imaginary, and real and loss tangent measurements), where *x* is the mean of three impedance measurements at a fixed frequency, μ the average of all the frequency ranges, and σ the standard deviation of all the frequency ranges. PCA was the choice of data proceeding to reduce the size of data and obtain a new space of orthogonal components, in which different concentration patterns can be observed and explained with Excel XLSTAT Programme. All of the sample analyses were carried out in duplicate or triplicate. 

## 3. Results and Discussion

### 3.1. Sensors Characterization: Morphology and Thickness

**Figure 2:**
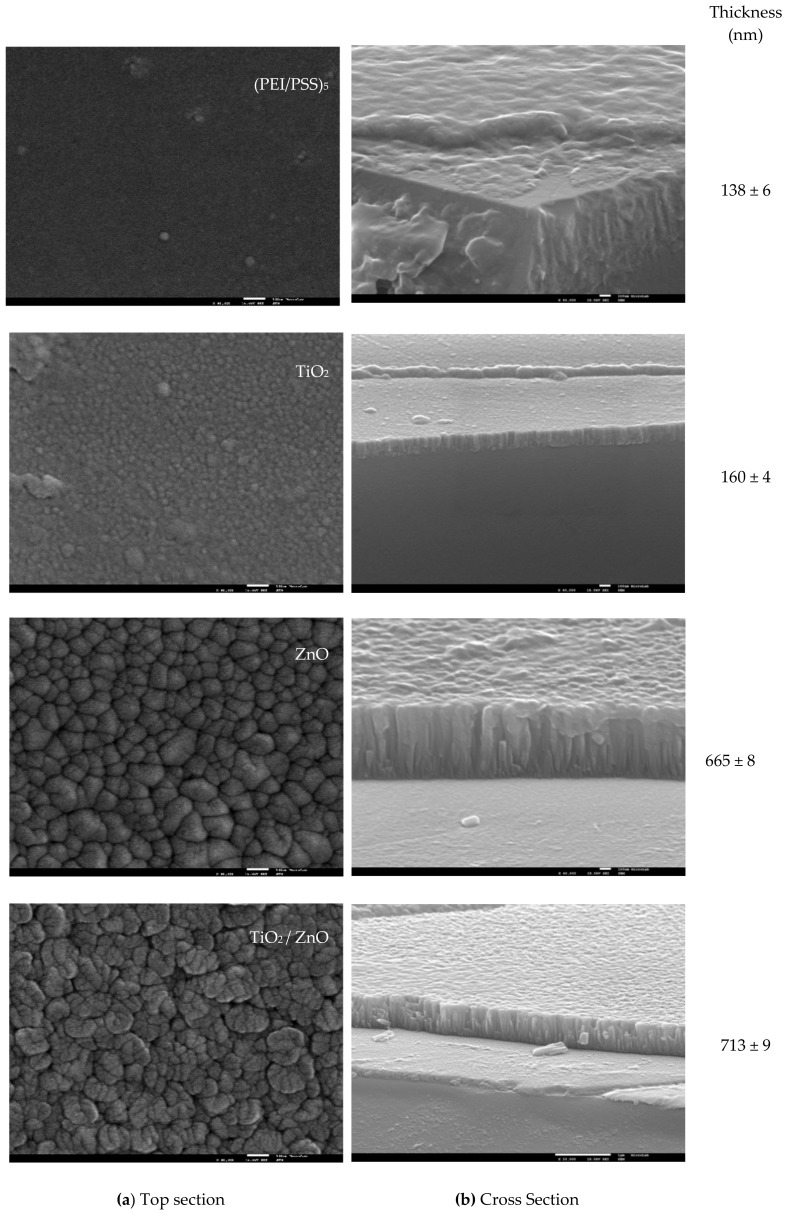
Scanning electron microscope (SEM) image of (PEI/PSS)_5,_ TiO_2_, ZnO and TiO_2_/ZnO thin films combinations: (**a**) Top and (**b**) cross section.

As described in the Materials and Methods section, (PEI/PSS)_5_ LbL films and TiO_2_, ZnO, and TiO_2_/ZnO sputtered films (see sensors composition in Figure S1 in Supplementary Materials) were prepared and characterized. Figure 2a,b presents the SEM images and the respective thickness of those thin films. The characterization of the sensors layers is important to explain the sensors performance, since the homogeneity, particle size, and thickness will affect the electrical measurements: resistance, molecules’ adsorption, capacitance, and further capability of detection.

In agreement with Figure 2a, glass conducting substrates are covered by homogeneous films. However, the surface morphology of these films changes according to the deposition technique. Thus, the LbL films exhibited a smooth surface with evidence of very small agglomerates or grains. On the contrary, sputtering films presented a rougher surface and much larger agglomerates with different shapes, depending on the oxide used. In particular, for TiO_2_ films, the agglomerates of grains with an average lateral size of 30 nm are distributed over the substrate surface with a ‘blooming flower-like’ appearance. In the case of ZnO films, the surface presents a pronounced cone-like morphology and lateral average size of approximately 100–120 nm. The outermost ZnO layer of the TiO_2_/ZnO bilayered films show globular shape agglomerates, with the lateral average size approximately 100–150 nm. The formation of agglomerates or particulates are a product of individual nano-phase grains that exhibit different dimensions (ZnO > TiO_2_), which result in an increase of the active surface area and also promotes the formation of porous films. These characteristics are critical in the response device to EOCs detection. The films thickness was evaluated from FEG-SEM cross-section images and Figure 2b summarizes the obtained values. It can be observed that the thickness of the (PEI/PSS)_5_, TiO_2_, ZnO, and TiO_2_/ZnO increases from 133 to 713 nm, in the mentioned order.

### 3.2. E-Tongue Training for Emerging Organic Contaminants Recognition

To train the e-tongue device, it is first necessary to find the “calibration curves” for the individual compounds. For that, i.e. to analyze their degradation pathway, impedance spectra of the sensors when immersed in effluent spiked with the EOCs, at different concentrations, were acquired.

**Figure 3:**
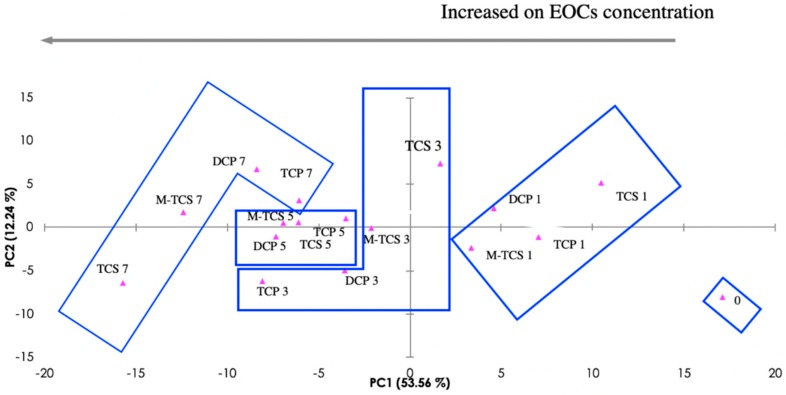
Principal component analysis (PCA) Score plot for the electronic tongue (five types of sensors) for 2,4,6-Trichlorophenol (TCP), 2,4-Dichlorophenol (DCP), triclosan (TCS), and metabolite methyl-triclosan (M-TCS)) individual measurements in the range of concentration 0, 1, 3, 5, 7, that correspond to the effluent as sampled, and effluent spiked with 0.1, 0.3, 0.5 and 0.7 mg emerging organic contaminants/L (EOCs/L).

The PCA method was applied to carry out an exploratory analysis of the obtained impedance data, though the reduction of the size of data and the creation of a new space of orthogonal components, in which different concentration patterns can be observed. Figure 3 presents the PCA score plot of the measured impedance data (see spectra in Figure S2 in the Supplementary Materials), obtained when the five sensors (composition on Figure S1 in Supplementary Materials; (PEI/PSS)_5_ onto 10 µm interdigitated electrode, (PEI/PSS)_5_ onto 200 µm interdigitated electrode, TiO_2_ onto 10 µm interdigitated electrode, ZnO onto 10 µm interdigitated electrode, and TiO_2_/ZnO onto 10 µm interdigitated electrode) were immersed in effluent that was spiked with the four EOCs at different concentrations. Correspondingly, the reproducibility of the measurements for the lowest concentrations (0.1 mg/L, more susceptible to error), for each sensor for each compound can be observed in Figure S3 (in Supplementary Materials).

On the PCA plot, as shown in Figure 3, the resulting analyte clustering by concentration are represented. The first two components of the PCA explain 65.80% of the observed variation. The plot observation supports the ability of the e-tongue device to “recognize” between individual EOCs-spiked effluents and raw effluent (non-spiked) matrices. A trend along the first component (PC1), according to the analyte concentrations might be observed on the plot, where the concentration increases from right to left across PC1 axis, moving away from concentration zero, and thus supporting the potential of the e-tongue device to discriminate quantitatively individual EOCs.

It was possible to determine individual “calibration curves” through the first two principal components data (Figure S4 in Supplementary Materials). These estimated curves are useful tools for a semi-quantitative extrapolation of the EOCs’ during EFR monitoring, where the effluent has a mix contamination. It was observed that the best fitting is not linear, as it is usually demanded and seen in chromatography methods [28], but polynomial, where the measure of goodness-of-fit observed ranged from R^2^ = 0.86 (TCP) to R^2^ = 0.99 (TCS).

### 3.3. E-tongue Performance for Emerging Organic Contaminants Semi-Quantification

**Figure 4:**
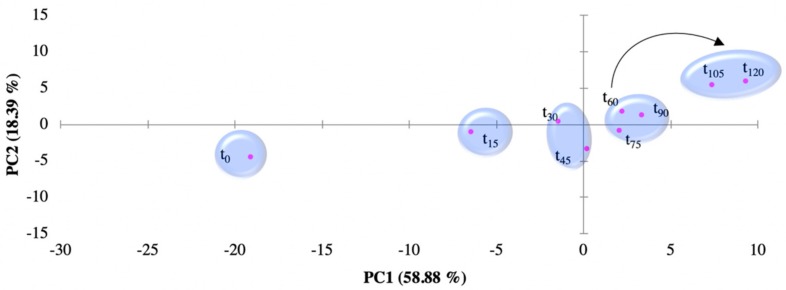
PCA Score plot for electrochemical flow reactor (EFR) degradation path measured by the electronic tongue in the effluent spiked with the four EOCs: t_0_ to t_120_ correspond to sampling every 15 min.

Figure 4 shows the PCA plot from the normalized impedance data that were obtained by the customized e-tongue for the EFR treatment dataset. The dataset is composed by the analysed aliquots that were collected every 15 min in the course of the EFR treatment (120 min; t_0_–t_120_). A pattern in the plot, through the two first principal components, related to the time of EFR treatment is identifiable. The first two components of the PCA explain 77.27% of the observed variation. This observable trend supports the device responsiveness towards the EOCs decreasing concentration (observation that is supported by HPLC data) across time. The trend follows a similar behaviour from that observed for the individual EOCs measurements, as on the mixed EOCs measurements are the highest concentrations that drift away from concentration zero (see Figure S5 in Supplementary Materials). As noted for the individual EOCs calibration plot (Figure 3), the e-tongue, when monitoring the mixed EOCS in the effluent (Figure 4), is also able to “recognize” between EOCs-spiked effluent and raw effluent (non-spiked) matrices.

**Figure 5:**
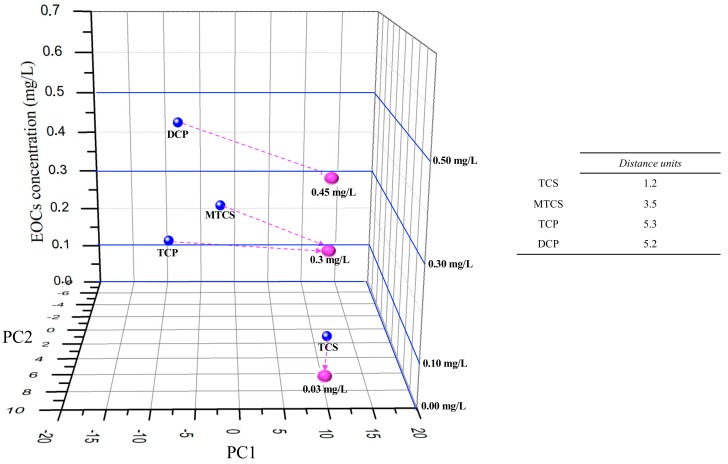
Visual evaluation of individual EOCs e-tongue semi-quantitative “sensorial signal” (individual vs mix measurements). Blue dots: EOCs Individual PC1 and PC2 data from Figure 3; Pink dots: high-performance liquid chromatography (HPLC) EOCs estimated concentrations for PC1 and PC2 data attained from Figure 4 at t_120._

A three-dimensional (3D) plot was developed (Figure 5), by adding to the PC1 and PC2 data axis, a Z axis with the EOCs concentration values t_0_ cross-check the individual EOCS e-tongue semi-quantitative “sensorial signal” (Figure 3) with the mixed EOCs “sensorial signal” at the end of EFR treatment (Figure 4). The purpose of adding the z axis was to allow for a visual evaluation of the concentration range dimension obtained for the EOCs at the end of EFR treatment. Three parameters compose the plot data: (1) the EOCs estimated concentrations by HPLC at t_120_ (TCP = 0.3 mg/L; DCP = 0.45 mg/L; TCS = 0.03 mg/L; and, M-TCS = 0.29 mg/L) and (2) the PC1 and PC2 data attained from Figure 4 at t_120_ (pink dots); (3) the interpolated PC1 and PC2 values from the data in Figure 3 that are close to the HPLC estimated concentrations (TCP = 0.3 mg/L; DCP = 0.5 mg/L; TCS = 0.0 mg/L; and, M-TCS = 0.3 mg/L) for each of the EOCs at t_120_ (blue dots).

It is observed on Figure 5 that, for TCS and M-TCS, the distance between the interpolation points and the calibration points was lower, when compared with DCP and TCP. It is important to refer that TCP and DCP are the most common by-products for the TCS and M-TCS degradation pathway, and thus they are created at the same time that they are degraded, which might explain the experimental results for these compounds.

Additionally, it was also important to understand which individual sensor, of the multi sensor e-tongue, had the higher influence in the impedance “sensorial signal” for each EOCs. Thus, for TCS and M-TCS, the best response was obtained with TiO_2_/ZnO, with the first two components of the PCA explaining 96.65% and 96.92% (see PCA data on Supplementary Materials, Figure S6a,b) of the total variance, respectively. For TCP, the best semi-qualitative analysis was achieved while using the TiO_2_ sensor, with the first two components of the PCA explaining 99.96% (see PCA data on Supplementary Materials, Figure S6c) of the total variance. Finally, for DCP, and apart from the others three EOCs, the (PEI/PSS)_5_ sensor with 10 µm interdigitated gold electrode had the highest semi-qualitative response with the first two components of the PCA, explaining 98.68% (see PCA data on Supplementary Materials, Figure S6d) of the total variance.

**Figure 6:**
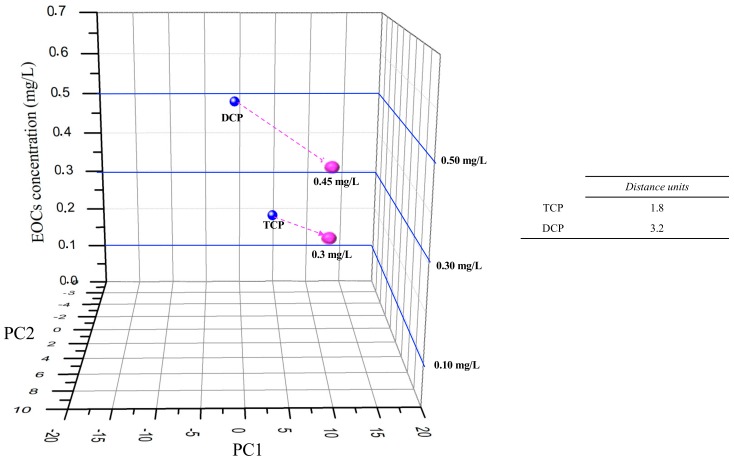
Visual evaluation of individual semi-quantitative “sensorial signal” (individual vs mix measurements) for TCP with TiO_2_ sensor and for DCP with (PEI/PSS)_5_ sensor. Blue dots: EOCs individual PC1 and PC2 data for TCP with TiO_2_ sensor and for DCP with (PEI/PSS)_5_ sensor; Pink dots: HPLC EOCs estimated concentrations for PC1 and PC2 data attained from Figure 4 at t_120_.

Figure 6 presents the same methodology for TCP and DCP, but only with TiO_2_ or (PEI/PSS)_5_ sensor impedance data for the semi-quantitative analyse of TCP and DCP.

Hence, analysing Figure 6, the distance between the interpolation points and the calibration points was lower (distance units decreased 3.2 to DCP and 1.8 to TCP), while comparing to Figure 5 e-tongue multisensory analyses for DCP and TCP.

The experimental data points to the importance of the nanomaterial used to build the e-tongue that will influence the impedance response as the thin film final morphology, structure, and properties of the thin films, and their consequent interactions with the different EOCs’ physical-chemical behaviour on the effluent matrix [29].

According to [30] reported data, the thickness of the film is related to the hydrophobic or hydrophilic final character. Thus, the film presents a more hydrophobic character when the thickness is higher. The thin film morphology of the tested sensors, as presented at Figure 2a, suggests a hydrophilic character for all the films tested, although, as the thickness increases, the hydrophobic characters also increase (Figure 2a, (PEI/PSS)_5_ < rough surface < TiO_2_/ZnO). According to the experimental data, the thin films with more “hydrophobic character”, due to film thickness, such as the TiO_2_/ZnO and ZnO thin films presented better impedance responses in the semi-quantitative analysis of TCS and M-TCS, the analytes with higher Log k_ow_ (Table S1 in Supplementary Materials).

For the sensors that were considered to be “more hydrophilic”, the TiO_2_ and (PEI/PSS)_5_ films presented the best responses for DCP and TCP, the less lipophilic analytes. To DCP, for instance, the higher porous size in the films with higher thickness, endorsed the molecule “semi-permeability” through the thin film net, “giving” to the molecules a fluid character, which may be masking their detection. Thus, in the TiO_2_/ZnO and ZnO thin films (“hydrophobic character”), the phenolic molecules movement will be faster than the detection, not allowing for the complete analysis understanding. In the polyelectrolytes thin film, (PEI/PSS)_5_, this behaviour does not occur, since the film layers are organic and without hole structures, only providing an impedance response to the electronegativity of the DCP and TCP, as the film combination is itself negatively charged.

## 4. Conclusions

At an electrochemical reactor’s outlet a customized e-tongue was attached, which was composed of five sensors, built up with layer-by-layer and sputtering thin-films, to access the degradation monitoring of TCS, M-TCS, DCP, and TCP. Three main issues were evaluated to analyse this hypothesis: (1) characterization of the thin-films; (2) e-tongue training for the recognition of the different EOCs; and, (3) e-tongue array performance in the detection and semi-quantification of the EOCs in a mix contaminated effluent. Therefore, performing impedance measurements into the e-tongue array, was observed analytes clustering by concentration in the calibration curves, where the first two components of the PCA explained 65.80% of the total variance. The e-tongue array showed responsiveness towards the EOCs decreasing concentrations during the electrochemical treatment, showing a pattern trend through the first two principal components, being explained by 77.27% of the total variance. The e-tongue recognized between individual EOCs-spiked effluents and raw effluent (non-spiked) matrices. At the end of the treatment, t_120_, the cross-check of the analytes individual semi-quantification was achieved for TCS and M-TCS with the e-tongue array. For DCP and TCP, better results were accomplished with a single sensor, (PEI/PSS)_5_ with 10 µm interdigitated gold electrode and TIO_2_, respectively. The characterization of thin films, while using field emission scanning electron microscope, allowed for a total understanding of the “sensorial” impedance response. The present study suggests innovative alternatives for complementing the traditional analysis with sensors devices, since the customized e-tongue was capable of semi-qualitative analyses thought electro-dynamic degradation’s kinetics.

## Supplementary Materials

The following are available online at https://www.mdpi.com/1424-8220/19/24/5349/s1, Figure S1. Scheme of the thin films used in the electronic tongue array; Figure S2. Resistance impedance spectra of each sensor device under study to TCP; DCP, TCS and M-TCS; Figure S3. Resistance impedance spectra of each sensor device under study to the effluent spiked with 0.1 mg/L of TCP; DCP, TCS and M-TCS (Reproducibility of n = 3 impedance measurements); Figure S4. Electronic tongue calibration curves for each EOCs under study: TCP, DCP, TCS and M-TCS, in the range of concentration: 0-0.8 ppm (x axis): data from Figure 3, first component PC1 (y axis); Figure S5. EFR degradation path measured by an electronic tongue in the effluent with the 4 EOCs: 0 mg/L (non-spiked; t_0_ to t_120_ (spiked with the 4 EOCs) sampled every 15 min; Figure S6. PCA Score plot for the best sensors, concerning effluent spiked (0–0.7 mg/L) with the 4 EOCs. Table S1. Chemical properties of the EOCs under study; Table S2. Average and standard deviation error to the electronic tongue presented at Figure 4.
