# Supplementary material for: Electronic Tongue Coupled to an Electrochemical Flow Reactor for Emerging Organic Contaminants Real Time Monitoring
Source: Sensors (Basel). 2019 Dec 4;19(24):5349. doi: 10.3390/s19245349 (PMC6960797; doi:10.3390/s19245349)
Supplement: Supplementary file 1 [file sensors-19-05349-s001.zip › Supplementary Data .pdf]

Supplementary Data

Table S1. Chemical properties of the EOCs under study.

Compound	**2,4,6-Trichlorophenol**	**2,4-Dichlorophenol**	**Triclosan**	**Methyl triclosan**
Structure	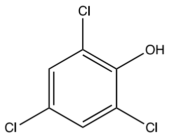	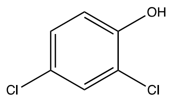	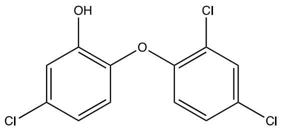	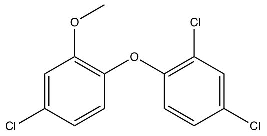
Formula	$C_6H_3Cl_3O$	$C_6H_4Cl_2O$	$C_{12}H_7Cl_3O_2$	$C_{13}H_9Cl_3O_2$
Molecular weight (g/ mol)	197.4	163	289.5	303.6
Solubility (mg/L) at 20 °C	500 *	4500	10	0.4
pKa[1]	6.23	7.8	7.9	-
Log K_{ow}[2]	3.69	3.06	4.76	5.0
k_H (atm/mol at 25°C)[3]	4.2 x 10^{-6}	4.29 x 10^{-6}	2.1 x 10^{-8}	-

[1]Ionization constant; [2] Logarithm of the octanol-water partition coefficient; [3] Henry's Law constant; *25°C

References: http://pubchem.ncbi.nlm.nih.gov/; https://chemicalize.com/

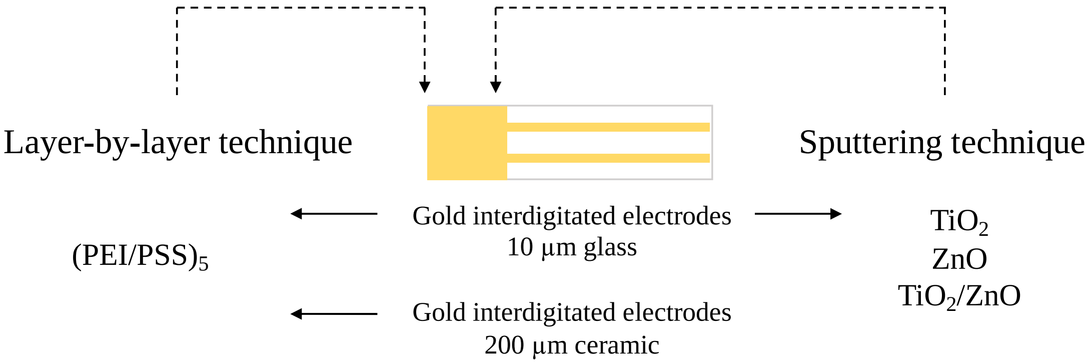

Figure S1. Scheme of the thin films used in the electronic tongue array.

	2,4,6-Trichlorophenol	2,4-Dichlorophenol	Triclosan	Methyl triclosan
(PEI/PSS)₅ with glass subtract 10 µm				
(PEI/PSS)₅ with ceramic subtract 200 µm				
TiO₂				

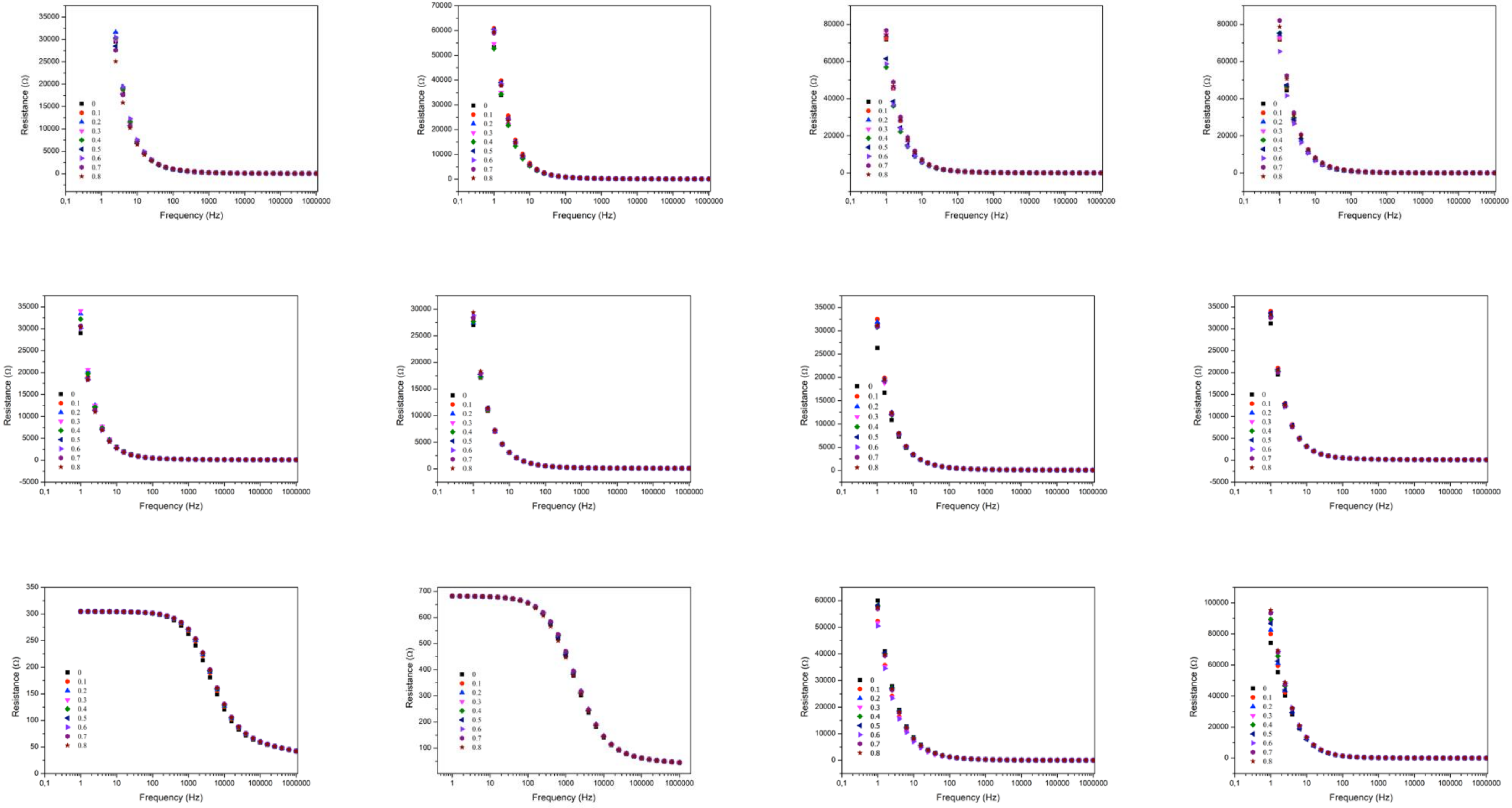

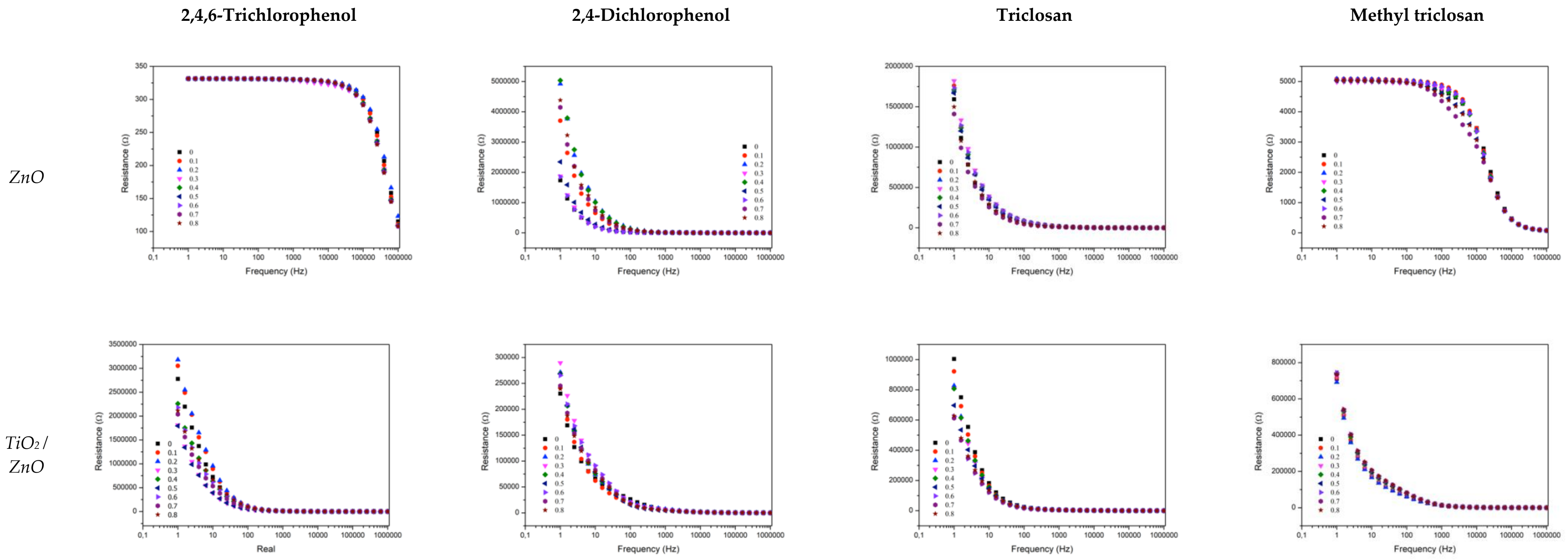

Figure S2. Resistance impedance spectra of each sensor device under study to TCP; DCP, TCS and MTCS.

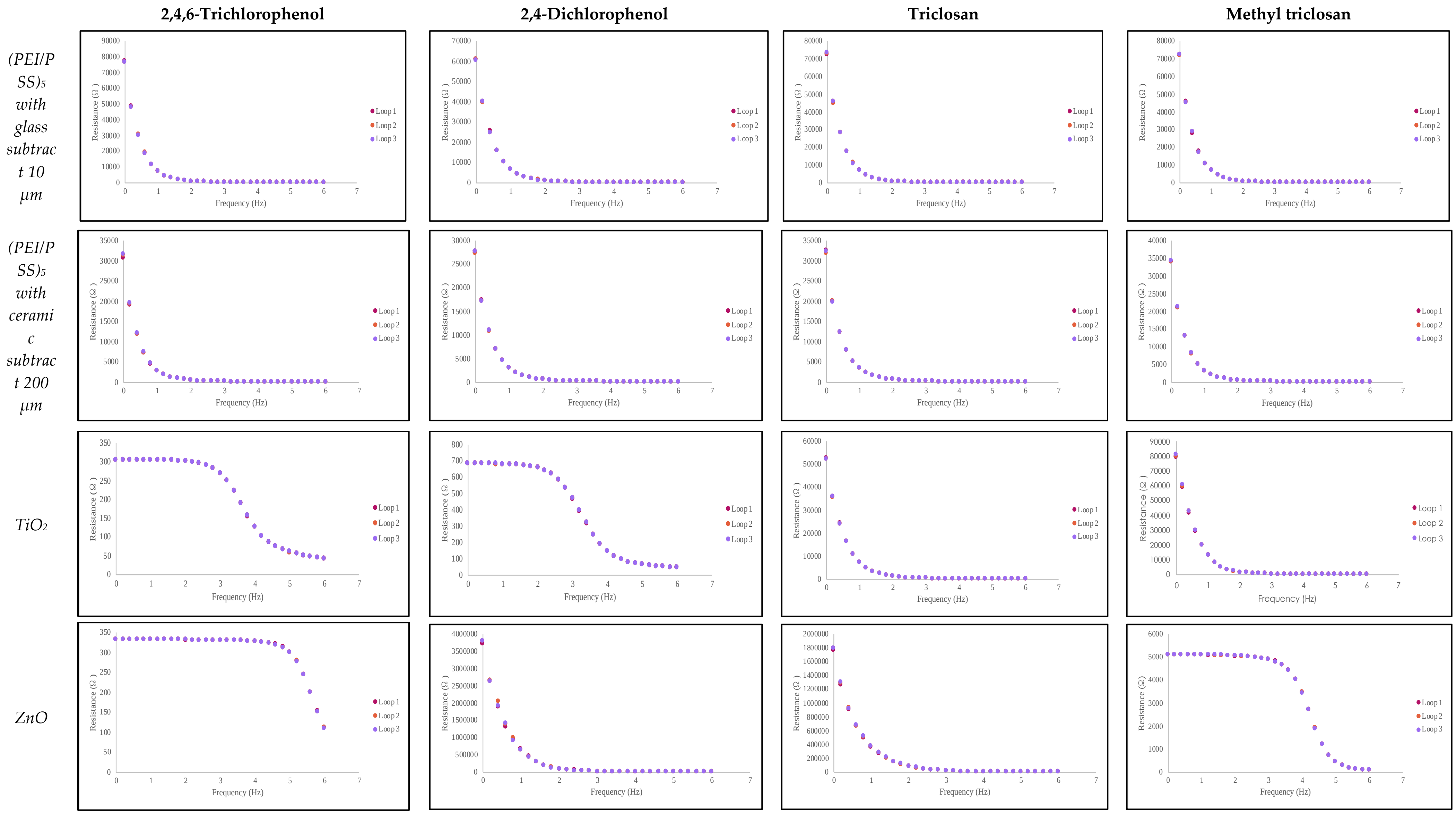

2,4,6-Trichlorophenol
2,4-Dichlorophenol
Triclosan
Methyl triclosan
(PEI/PSS)5 with glass subtract 10 μm
(PEI/PSS)5 with ceramic subtract 200 μm
TiO2
ZnO
Resistance (Ω)
Frequency (Hz)
Loop 1
Loop 2
Loop 3

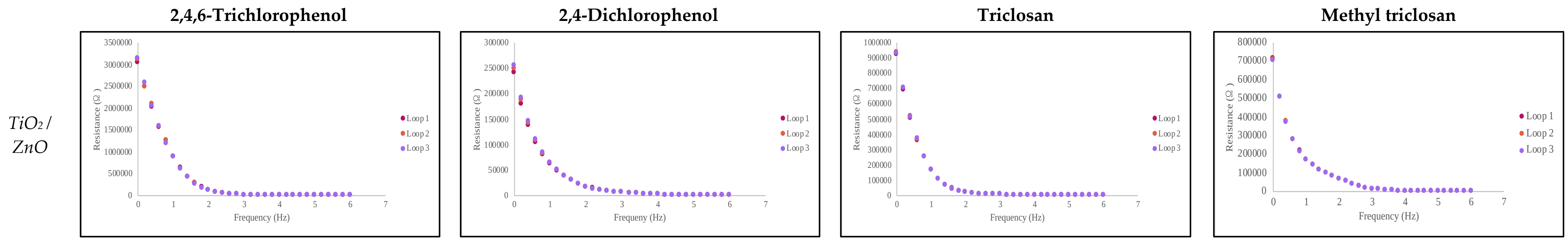

Figure S3. Resistance impedance spectra of each sensor device under study to the effluent spiked with 0.1 mg/L of TCP; DCP, TCS and MTCS (Reproducibility of n=3 impedance measurements).

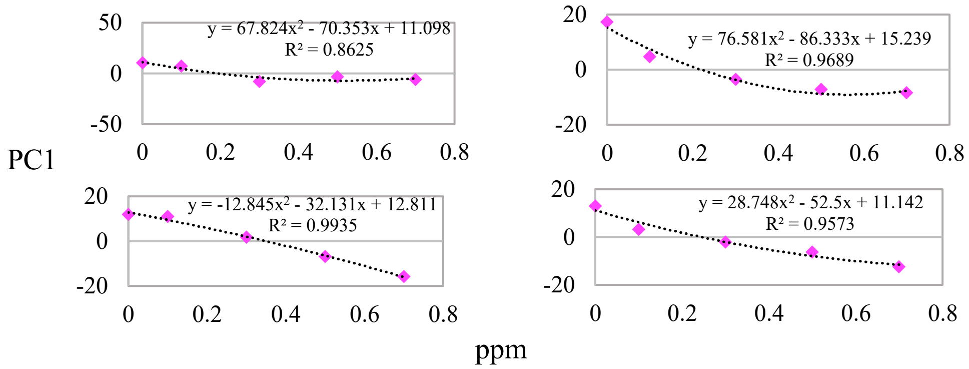

Figure S4. Electronic tongue calibration curves for each EOCs under study: TCP, DCP, TCS and M-TCS, in the range of concentration: 0-0.8 ppm (x axis): data from Figure 3, first component PC1 (y axis).

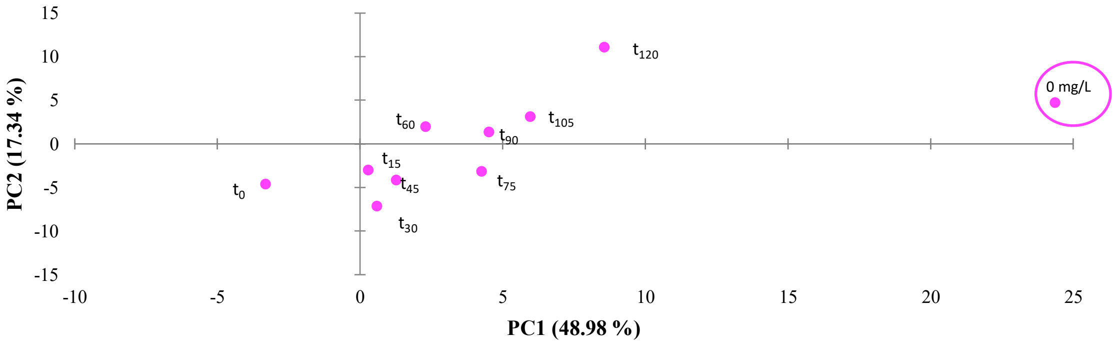

Figure S5. EFR degradation path measured by an electronic tongue in the effluent with the 4 EOCs: 0 mg/L (non-spiked; t_0 to t_{120} (spiked with the 4 EOCs) sampled every 15 min.

Table S2. Average and standard deviation error to the electronic tongue presented at Figure 4.

	**PC1**		**PC2**	
Sampling every 15 min *	$\underline{X}$	**SD**	$\underline{X}$	**SD**
t_0	-19.1	4.9	-4.4	5.6
t_{15}	-6.5	4.8	-1.0	4.9
t_{30}	-1.5	1.7	0.4	3.7
t_{45}	0.1	4.1	-3.3	4.9
t_{60}	2.2	0.1	1.9	0.3
t_{75}	2.0	1.6	-0.7	2.0
t_{90}	3.3	0.8	1.3	0.4
t_{105}	7.3	1.1	5.5	1.9
t_{120}	9.3	0.8	5.9	3.7

N= 2; X – Average; SD – standard deviation

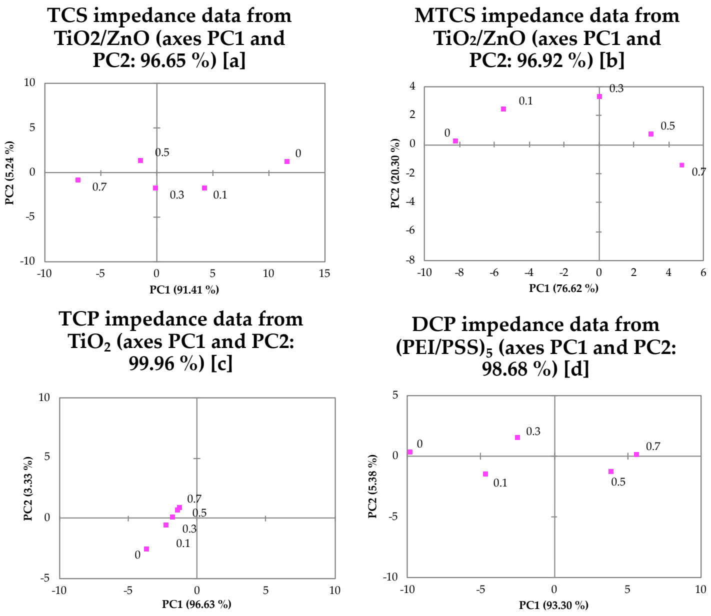

Figure S6. PCA Score plot for the best sensors, concerning effluent spiked (0-0.7 mg/L) with the 4 EOCs.
